# Roles of Mesenchymal Stem Cell-Derived Exosomes in Cancer Development and Targeted Therapy

**DOI:** 10.1155/2021/9962194

**Published:** 2021-07-10

**Authors:** Zixuan Sun, Jiaxin Zhang, Jiali Li, Mi Li, Jing Ge, Peipei Wu, Benshuai You, Hui Qian

**Affiliations:** Zhenjiang Key Laboratory of High Technology Research on Exosomes Foundation and Transformation Application, School of Medicine, Jiangsu University, Zhenjiang 212013, China

## Abstract

Exosomes have emerged as a new drug delivery system. In particular, exosomes derived from mesenchymal stem cells (MSCs) have been extensively studied because of their tumor-homing ability and yield advantages. Considering that MSC-derived exosomes are a double-edged sword in the development, metastasis, and invasion of tumors, engineered exosomes have broad potential use. In this review, we focused on the latest development in the treatment of tumors using engineered and nonengineered MSC-derived exosomes (MSC-EXs). Nonengineered MSC-EXs exert an antitumor effect on several well-studied tumors by affecting tumor growth, angiogenesis, metastasis, and invasion. Furthermore, engineered exosomes have promising research prospects as drug-carrying tools for the transport of miRNAs, small-molecule drugs, and proteins. Although exosomes lack uniform standards in terms of definition, separation, and purification, they still have great research value because of their unique advantages, such as high biocompatibility and low toxicity. Future studies on MSC-EXs should elucidate the mechanisms underlying their anticancer effect and the safety of their application.

## 1. Introduction

The diagnosis and treatment of tumors, which are a key global health concern, have long been a focus of research. Although surgery is still an effective strategy for early cancer treatment, owing to the hidden onset of cancer and the limitations of diagnostic techniques, most patients with cancer have already reached intermediate and advanced tumor stages by the time their cancers are detected, minimizing the therapeutic effect of surgery [1]. Although emerging immunotherapy has been successfully applied, the heterogeneity of tumors hinders the wide application of immunotherapy for all tumors [2, 3]. Hence, radiotherapy and chemotherapy are still the preferred methods of treating tumors. However, drug delivery issues and drug resistance have become major challenges related to chemotherapy and radiotherapy.

MSC-EXs, which are natural nanovesicles with low immunogenicity, good biocompatibility, and low cytotoxicity, have a unique antitumor effect. As MSC-EXs are high-quality drug delivery carriers, constructing engineered MSC-EXs for drug loading has long been a key research topic in the field of exosome-based therapy for cancer. Moreover, natural exosomes that function through their own inclusions or surface markers also exert antitumor effects; some of them may be used as biomarkers for cancer diagnosis or prognosis [4–7]. Given that the role of natural exosomes in tumors is still controversial, in this study, we reviewed both the positive and negative effects of natural MSC-EXs on tumor occurrence and development.

## 2. Biogenesis and Uptake of Exosomes

Extracellular vesicles (EVs) are classified as apoptotic bodies (500 nm to 2 *μ*m), microvesicles (MVs; 100 to 1000 nm), and exosomes (30 to 150 nm) mainly based on their diameter and origin [8]. MVs, also known as exfoliated vesicles or extracellular bodies [9, 10], germinate from the plasma membrane, and apoptotic bodies are formed by bubbles in the apoptotic cell membrane [11].

Exosomes are formed from the double invagination of the plasma membrane [12]. The plasma membrane that invades inward for the first time invaginates to form early endosomes which germinate inward to form intraluminal vesicles (ILVs) [9, 12, 13]. Two mechanisms are behind the formation of ILVs: the pathway mediated by the endosomal sorting complex required for transport (ESCRT) and the ESCRT-independent pathway [14, 15]. The ESCRT is the most studied mechanism [16]. The formed ILVs can selectively capture certain molecules in the cell [17]. The composition of exosomes is highly heterogeneous. This feature is related to the cell shape, stimulation state, stress state, metamorphosis, and differentiation function of the original cell type. This shows that content such as miRNA and protein recruitment into exosomes may be a regulated process [11, 18]. Late endosomes containing ILVs are termed multivesicular bodies (MVBs) [9]. Some MVBs are transported to the trans-Golgi network for endosomal circulation and finally reach the lysosome to degrade all the carried substances [11]. Some MVBs are transported under the influence of the Rab GTPase family, cytoskeleton (microtubules and microfilaments), molecular motors (dynein and kinesin), and membrane fusion devices (SNARE complex) [19]. They fuse with the plasma membrane and eventually release exosomes.

**Figure 1:**
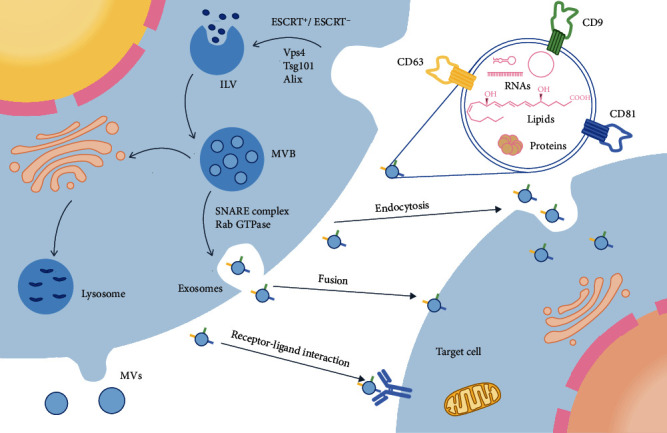
Biogenesis and cellular uptake of exosomes. The plasma membrane invaginates through the ESCRT-dependent and ESCRT-independent pathways to form ILVs. Late endosomes containing ILVs are called multivesicular bodies (MVBs). Some MVBs are transported to the Golgi complex circulation and finally transported to the lysosome for degradation, and some MVBs are fused with the plasma membrane under the influence of the Rab family, SNARE complex, and tubulin before being released from the cell. Exosomes released from cells can enter target cells through fusion, receptor-mediated endocytosis, macrophage phagocytosis, or phagocytosis. The surfaces of exosomes contain many molecules. This figure shows three common molecules (CD9, CD63, and CD81) on the surface of MSC-EXs.

Target cells uptake exosomes mainly through fusion, receptor-mediated endocytosis, macrophage phagocytosis, or phagocytosis [16]. In addition, a study reported that cells preferentially absorb smaller exosomes, especially those with diameters of 40–50 nm [9]. The uptake of exosomes depends not only on the nature of exosomes but also on the type and physiological state of the recipient cells [9] (Figure 1).

## 3. Characterization of Exosomes

The surfaces of exosomes contain various markers. At present, 9769 proteins have been identified in exosomes from different sources, such as CD9, CD63, CD81, Alix, EP-CAM, Hsp70, Tsg101, CD55, and CD59 [20]. Studies have reported that all MSC-EXs express markers CD9, CD63, and CD81 [21, 22]. CD55 and CD59 help avoid the activation of opsonin and coagulation factors, thus stabilizing the distribution of exosomes in biological fluids [23]. In addition, CD9, CD63, Alix, and EP-CAM can be used for the isolation of exosomes. Magnetic beads, which play a central role in capture-based techniques, are a new type of tool that can be modified to bind to target proteins on the membrane surface. CD9, CD63, Alix, and EP-CAM can be enriched by antibody-coated magnetic beads. According to the binding of antibody-coated magnetic beads to the target protein on the membrane surface, the process of collecting immobilized specific exosomes can be achieved by washing in the stationary phase [24].

## 4. Direct and Indirect Effects of MSC-EXs on Tumors

Extensive evidence has confirmed that MSC-EXs play a key role in angiogenesis, tumor growth, and metastasis (invasion). Among the various sources of exosomes, human umbilical cord MSCs (hUC-MSCs) and human umbilical cord Wharton's jelly MSCs (hWJ-MSCs) are the most commonly used. Whether natural MSC-EXs exert positive or negative effects on tumors is still controversial. A previous study reported that the dual effect seems to be related to the source of MSC-EXs, dose, and time of MSC injection, cancer type, and other factors [25].

### 4.1. Dual Effects of MSC-EXs on Tumors

A group of studies has reported that natural MSC-EXs promote tumor progression [26]. However, some reliable evidence still indicates that MSC-EXs can inhibit the occurrence and development of tumors.

#### 4.1.1. Angiogenesis

Abnormal and excessive angiogenesis during cancer development could exacerbate the disease [27]. In the tumor microenvironment, MSC-EXs can enhance intracellular communication and promote angiogenesis [28]. The mechanism for promoting angiogenesis could be enhancing the tube-forming ability of endothelial cells (ECs) via exosomes. Evidence has suggested that microRNAs could play a key role in it. For example, Gong et al. [29] reported that the vascular precursor receptors (e.g., miR-30b) in MSCs derived from mouse embryos were transported to human umbilical vein endothelial cells (hUVECs) by MSC-EXs, which could directly promote the formation of the tube-like structure of hUVECs *in vitro*.

#### 4.1.2. Tumor Growth

MSC-EXs are a double-edged sword in tumor growth. Kalimuthu et al. [30] used flow cytometric analysis of FITC-Annexin V staining to assess the apoptotic effects of EV treatment and confirmed that MSC-derived extracellular vesicle (MSC-EV) treatment induced apoptosis in Lewis lung carcinoma cells. By contrast, Huang et al. [31] reported that exosomes derived *in vitro* from bone marrow MSCs (BM-MSCs) could enhance tumorigenesis by promoting oncogenic autophagy in osteosarcoma.

#### 4.1.3. Metastasis and Invasion

Tumor metastasis and invasion are regulated not only by the cancer cells themselves but also by the entire tumor microenvironment. As a part of the tumor microenvironment, the role of exosomes in information transmission cannot be ignored. Gu et al. [32] observed that exogenous hUC-MSC-derived exosomes (hUC-MSC-EXs) could facilitate the growth and migration of gastric cancer cells by activating the Akt pathway. In addition, endogenous MSC-EVs derived from murine and human bone marrow can induce breast cancer cells to enter the bone marrow and survive as cancer stem cells (CSCs) in a dormant state for decades [33].

### 4.2. Influence of MSC-EXs on the Growth and Development of Different Types of Tumors

Considering the heterogeneity of tumors, different tumor types may be among the reasons for the seemingly contradictory effects of MSC-EXs.

#### 4.2.1. Effects on Breast Cancer

The incidence of breast cancer has been increasing in recent years [34]. According to statistics by Miller et al., breast cancer was still the most common cancer among women in the United States as of 2019, and it may affect at least 1,000,000 women by 2030 [35]. The promotional effect of MSC-EXs on tumors has been largely confirmed. Furthermore, MSC-EXs also have an inhibitory effect on tumors. Research has shown that exogenous MSC-EXs activate the extracellular signal-regulated kinase pathway to promote the proliferation and migration of breast cancer cells *in vitro* [36]. In addition, as exosomes derived from adipose-derived MSCs (ADSCs) *in vivo* contain various miRNAs that regulate epithelial-mesenchymal transition (EMT), they can promote breast cancer cells to enter the dormant phase, which is related to higher chemoresistance [37]. Pakravan et al. [38] found that BM-MSC-derived exosomes (BM-MSC-EXs) *in vitro* are rich in miR-100, which modulates the mTOR/HIF-1*α*/VEGF signaling axis and inhibits the angiogenesis of breast cancer (Table 1).

#### 4.2.2. Effects on Multiple Myeloma

Multiple myeloma (MM) accounts for about 10% of all hematological malignancies, with an overall survival in affected patients of only 3 years [43]. Extensive research has been conducted in this regard. Roccaro et al. [44] found that homogeneous MSC-EXs from different sources exerted completely different effects on MM. Interestingly, Umezu et al. [45] found that young donor-derived BM-MSC-EXs *in vitro* could be more competent in inhibiting MM-induced angiogenesis than BM-MSC-EXs from old donors and could thus improve the overall survival of patients.

#### 4.2.3. Effects on Gastric Cancer

Gastric cancer (GC) is one of the most common malignancies worldwide [46]. Chemotherapy is still the main treatment for GC. Further, the approach that inhibits human epidermal growth factor receptor 2 is clinically proven to significantly improve survival rates [47]. Nevertheless, the treatment and prognosis of these patients are still poor. The research of exosomes brings new possibilities, which can be further applied to the discovery, treatment, and prognosis of cancers (Table 2).

#### 4.2.4. Effects on Liver Cancer

Extensive research has shown that MSCs and MSC-EXs have considerable potential for the treatment of liver cancer. MVs derived from BM-MSCs inhibit cell cycle progression and induce apoptosis in HepG2 cells *in vitro*. Moreover, intratumor administration of BM-MSC-derived MVs *in vivo* remarkably inhibited tumor growth [50]. hUC-MSC-EXs containing miR-451a can limit the EMT of hepatocellular carcinoma (HCC) cells by targeting ADAM10; this may provide a new target for HCC therapy [51].

#### 4.2.5. Effects on Bladder Cancer

In recent years, the morbidity and mortality of bladder cancer have had an evident upward trend, making bladder cancer among the most common urinary system malignancies. Exosomes and the engineered ones are of great importance to bladder cancer patients for their future research and treatment. The inhibitory effect of MSC-EXs on bladder cancer is greater than that on other major tumors. Cai et al. [52] investigated the effect of BM-MSC-EXs on bladder cancer cells through loss- and gain-of-function experiments. The experimental results showed that exosomal miR-9-3p upregulation could inhibit the expression of endothelial cell-specific molecule 1 (EMS1), thereby inhibiting the progression of bladder cancer. Some researchers have also studied the effect of MSC-derived exosomal miRNA on bladder cancer cells. Fu et al. [53] extracted BM-MSC-EXs for an miR-19b-1-5p inhibition and elevation test and found that BM-MSC-derived exosomal miR-19b-1-5p could inhibit the growth of bladder cancer by downregulating nonreceptor protein tyrosine kinase Arg (ABL2). Similarly, Jia et al. [54] found that hUC-MSC-derived exosomal miR-139-5p could inhibit the progression of bladder cancer through targeting and downregulating PRC1.

#### 4.2.6. Effects on Prostate Cancer

In cancer targeted therapy research, up- or downregulation of the expression of a certain molecule often arouses the interest of researchers, who then, through interference measures, attempt to reverse this phenomenon and observe whether it has clinical value. Che et al. [55] chose MSC-EXs to implement this intervention for prostate cancer, which is the second most common cause of cancer-related deaths among men in developed countries [56]. miR-143 expression is downregulated in prostate cancer cells, and TFF3 expression is upregulated. Through their own exosomes, MSCs can deliver overexpressed miR-143 to prostate cancer cells, and this downregulates TFF3 expression, thereby inhibiting the proliferation and invasion of prostate cancer cells and promoting their apoptosis. Similarly, Jiang et al. [57] initially found that miR-205 expression was downregulated and rhophilin Rho GTPase binding protein 2 (RHPN2) expression was upregulated in prostate cancer cells. The upregulated miR-205 inhibited the proliferation, invasion, and migration of prostate cancer cells and promoted apoptosis by targeting RHPN2, and this was effected through miR-205-expressing exosomes derived from human bone marrow mesenchymal stem cells.

#### 4.2.7. Effects on Ovarian Cancer

Ovarian cancer has the highest mortality rate among gynecological malignancies; its detection in early stages is challenging [58]. Even with advancements in medical technology, the standard treatments for ovarian cancer are still cytoreductive surgery and platinum-based adjuvant chemotherapy [58]. Research on MSC-EXs revealed their potential applicability in the treatment of ovarian cancer. Reza et al. [59] demonstrated that miRNA contained in exogenous exosomes derived from human ADSCs can effectively reduce the viability of A2780 and SKOV-3 ovarian cancer cells and inhibit their proliferation. Qiu et al. [60] stated that hUC-MSC-derived exosomal miR-146a can target LAMC2 to regulate the PI3K/Akt signaling pathway, thereby inhibiting the growth of ovarian cancer cells and their chemoresistance. Li et al. [61] observed that MSC-EVs overexpressing miR-424 can suppress hUVEC proliferation, migration, and tube formation by inhibiting MYB, thereby further inhibiting the proliferation, migration, and invasion of ovarian cancer cells. Together, these studies provide new insights into the prevention, treatment, and prognosis of ovarian cancer.

## 5. Engineered MSC-EXs for Cancer Treatment

Exosomes were originally considered cell cleaners for the disposal of unnecessary components [62–64]. However, their low immunogenicity and toxicity, long half-life, high biocompatibility, tumor-homing ability, and other advantages make them a high-quality drug delivery tool for cancer treatment [65]. In addition to traditional methods for constructing engineered exosomes such as cocultivation, electroporation, freezing and thawing, and mechanical extrusion, genetic engineering has become a more attractive option.

### 5.1. miRNAs: A Tumor Treatment Tool

Currently, targeted drug delivery for tumors has been investigated to target specific subcellular compartments, and receptor-mediated endocytosis is the most promising approach [66]. As promising tumor treatment tools [67], miRNAs are difficult to pass through cell membranes owing to their negative charge and hydrophilic nature. Moreover, they are easily degraded after entering the body. As high-quality carriers, exosomes can address this concern [68]. In related research, the exosomes of hUC-MSCs expressing miRNAs have been highlighted as important carriers for gene or drug therapy [69].

Gene modification is the most commonly used strategy for miRNA transfection of MSC-EXs [70]. For example, Wu et al. [71] used bone marrow mesenchymal exosomes overexpressing miR-126-3p in a coculture with pancreatic cancer cells and found that miR-126-3p inhibited the development of pancreatic cancer by targeting ADAM9. Similarly, Yuan et al. [69] cocultured MSC-EXs overexpressing miR-148b-3p with breast cancer cell line MDA-MB-231 and found that miR-148b-3p inhibited proliferation, invasion, and migration but promoted apoptosis in breast cancer cells by downregulating TRIM59. Another study reported that the exosomal miR-205 derived from hBM-MSCs delayed the progression of prostate cancer by inhibiting RHPN2 [57]. MSC-EXs enriched with miR-185 were expected to serve as a new treatment option for oral leukoplakia because they can reduce inflammation, inhibit cell proliferation and angiogenesis, and induce cell apoptosis [72]. Notably, *β*-catenin, a key molecule of the Wnt/*β*-catenin signaling pathway, plays an important role in tumor EMT. Wan et al. [73] first conducted a study on the inhibitory effect of miR-34c on *β*-catenin in nasopharyngeal carcinoma (NPC). They obtained exosomes overexpressing miR-34c by transfecting MSCs with lentivirus, and they found that exosomes overexpressing miR-34c considerably increased radiation-induced apoptosis in NPC cells. miR-34c reduced the expression of *β*-catenin by directly targeting the 3′-UTR region of *β*-catenin mRNA, which contributes to a reduction in EMT and radioresistance. In addition, Jeong et al. [68] used 2D and 3D microfluidic devices cultured with hUVECs and A549 cells to simulate the tumor-like microenvironment of non-small-cell lung cancer, and they demonstrated that the miR-497 exosomes could act synergistically on endothelial cells and tumor cells to inhibit tumor growth, migration, and angiogenesis. This indicates that the combination of exosome-mediated miRNA therapeutic technology and microfluidic technology could become a predictive tool for the development of tumor targeted therapy. Liang et al. [74] constructed tumor-derived exosomes carrying both 5-FU and miR-21 inhibitor oligonucleotide (miR-21i) through electroporation and lentiviral transfection. Compared with miR-21i or 5-FU alone, the combinational delivery of miR-21i and 5-FU effectively reversed drug resistance and remarkably enhanced the cytotoxicity of 5-FU-resistant colon cancer cells.

### 5.2. High-Quality Transportation System for Small-Molecule Drugs

Chemotherapeutic drugs can be loaded onto MSC-EXs for administration, which can help address concerns related to their low aqueous solubility and specificity; this loading can improve the effects of related cancer therapy [75].

Taxol is a widely used chemotherapy drug. Melzer et al. [76] treated MSC544 cells with Taxol, and then exosomes were isolated using a serum-free MSC544 medium under continuous centrifugation conditions after 24 h. Human MDA-hyb1 triple-negative breast cancer cells were injected subcutaneously to induce subcutaneous tumors in 15 NOD/SCID mice. Subsequently, Taxol-loaded MSC544 exosomes were injected intravenously into tumor-bearing mice. The results showed that Taxol-loaded MSC544 exosomes showed superior tumor-reducing abilities. Some researchers have investigated the possibility of encapsulating paclitaxel into exosomes derived from other cells. For example, Agrawal et al. [77] successfully loaded Taxol on milk-derived exosomes and effectively overcame the barriers of the low oral bioavailability and cytotoxicity of Taxol. Similarly, Han et al. [78] discovered that natural killer cell-derived exosome-encapsulated paclitaxel exerted antitumor effects by inducing the upregulation of Bax and caspase-3 in tumor cell apoptosis signaling pathways.

Some researchers have focused on doxorubicin (DOX), which is also a common chemotherapy drug. Wei et al. [79] mixed exosomes with DOX-HCl, desalted the mixture with triethylamine, and then dialyzed it with PBS overnight to prepare EXs (EX-DOX) containing adriamycin. They found that EX-DOX had a favorable therapeutic effect against osteosarcoma. Similar results were also observed in the mouse breast cancer models used by other researchers. They used electroporation to load DOX into exosomes derived from MSCs to treat breast cancer and observed that EX-DOX significantly reduced the growth rate of tumors in a mouse breast cancer model [80].

Honokiol is a newly discovered chemotherapy drug for tumor treatment. Kanchanapally et al. [81] loaded honokiol into MSC-EXs by the sonication method, and the results indicated that its antitumor effect was 4–5 times greater than that of free honokiol.

In addition to the application of single drugs, the combined use of tumor necrosis factor-related apoptosis-inducing ligand (TRAIL) and other drugs is also a popular research direction for tumor treatment. Qiu et al. [82] obtained MSCT-EXs/CTX by combining CTX (a taxane drug) with TRAIL for the treatment of oral squamous cell carcinoma. The delivery system showed good antitumor activity *in vitro*; it effectively reversed the multidrug resistance of tumors and improved the sensitivity of chemotherapy drugs. In another study, researchers obtained CXCR4/TRAIL-rich exosomes from MSCs overexpressing both CXCR4 and TRAIL, and these exosomes acted synergistically with carboplatin (a first-line drug for the treatment of metastatic breast cancer) to exert antibreast metastasis effects *in vivo* [83].

Drug resistance has reduced the efficacy of chemotherapy and radiotherapy, which are first-line cancer treatments [75]. Therefore, a new generation of tumor treatment methods, including EVs, immunotherapy, and nanotechnology, is being gradually developed [75, 84]. Currently, MSCs are the only known cells capable of producing exosomes on a large scale [75, 83, 85]. Thus, MSC-EXs seem to be the most promising carriers for delivering specific drugs to tumor cells.

### 5.3. Research Difficulties of Engineered Exosomes: Proteins

The cargos carried by engineered exosomes are mainly short RNA sequences, small-molecule drugs, and proteins [86]. Few studies have reported the successful loading of proteins into exosomes [87, 88]. This may be attributable to the higher molecular weight of proteins, unclear mechanism of protein sorting by exosomes, and lack of related loading methods.

Mizrak et al. [89] first reported protein loading into EVs for antitumor therapy. Sterzenbach et al. [87], who were inspired by the release of enveloped viruses, developed a new method for loading proteins into exosomes based on the evolutionarily conserved late-domain (L-domain) pathway. Using this new method, they demonstrated that Cre recombinase labeled with a WW tag could be ubiquitinated and loaded into exosomes after being recognized by the L-domain-containing protein Ndfip1.

As previously mentioned, Liu et al. [83] were the first to use TRAIL in combination with CXCR4. CXCR4 is the most common chemokine receptor in human cancer cells. TRAIL can induce apoptosis in various cancer cells. Liu et al. transfected MSCs with CXCR4 and TRAIL through lentiviral transfection to obtain Exo^CXCR4+TRAIL^, and they observed that Exo^CXCR4+TRAIL^ exerted a significant synergistic effect with carboplatin in the mouse model.

**Figure 2:**
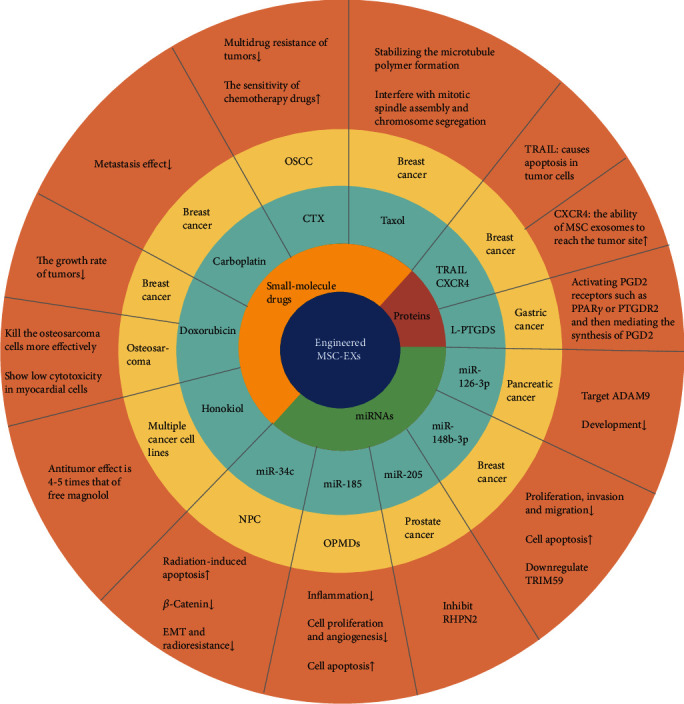
Engineered MSC-EXs for cancer treatment. The substances used for tumor treatment carried by the engineered MSC-EXs mainly include miRNAs, small-molecule drugs, and proteins. Four recent studies provide detailed information about miRNA loading in engineered MSC exosomes. Small-molecule drugs mainly include paclitaxel, CTX, carboplatin, doxorubicin, and magnolol. Compared with miRNAs and small-molecule drugs, few studies have reported the successful loading of proteins into exosomes.

These results indirectly indicate that extensive research with regard to proteins is warranted. For instance, a clinical case showed that the expression of lipocalin-type prostaglandin D synthase (L-PTGDS) in GC tissue was significantly reduced, and low expression of L-PTGDS was associated with shorter patient survival time [90]. Overexpression of L-PTGDS in GC cells inhibited their growth, clone formation, and migration ability. This suggested that L-PTGDS could inhibit the progression of GC and could be a potential therapeutic molecule for GC treatment. L-PTGDS likely exerts its antitumor effect by mediating the synthesis of PGD2 and activating PGD2 receptors such as PPAR*γ* or PTGDR2 (prostaglandin D2 receptor 2) on the surface of tumor cells, thereby inhibiting the malignant progression of tumors. By combining the carrier advantages of MSC-EXs and the antitumor effect of L-PTGDS, we applied genetic engineering technology to construct MSC-EXs carrying L-PTGDS to inhibit the malignant progression of GC in order to provide new ideas and methods for the biological treatment of GC (Figure 2).

## 6. Conclusions and Prospects

Since Rothman, Schekman, and Sudhof won the Nobel Prize in 2013 for revealing the transport regulation mechanism of intracellular vesicles such as exosomes, perspectives on exosomes have finally transformed; they were once considered to be “cell cleaners” but are now taken seriously in the field of scientific research.

Although the role of natural MSC-EXs in cancer is still controversial and a group of studies has proved that MSC-EXs play a role in promoting cancer progression, their tumor suppressor effects have also been reported. However, some scholars attribute these antitumor effects to differences in the materials and methods used in these studies, indicating that their effects are not universal.

In addition to the functions of surface markers and the content of MSC-EXs, MSC-EXs themselves are also high-quality drug delivery carriers. Further research is required to explore the role of MSC-EXs in cancer development and treatment and to identify solutions to the following challenges. First, the identification of exosomes lacks uniform international standards. Second, the low yield and high cost of this process limit its application. Third, traditional centrifugation methods require a long time to extract exosomes, and existing kits are expensive; moreover, the purity of the isolated exosomes is not ideal. Finally, because the quality of exosomes is greatly affected by temperature and time, the storage of exosomes is also challenging [91]. In short, MSC-EXs have high potential for use in cancer treatment, but they need to solve the problems of using themselves as tools first.

## Figures and Tables

**Table 1:** Effects and mechanisms of MSC-EXs on breast cancer.

Source	Effect	Mechanism	Model	Ref
ADSC	Stimulating metastasis of BCC	Type 2 diabetes mellitus altered the functions of MSC-EVs	*In vivo*	[39]
ADSC	Reduced tumor cell proliferation and migration and enhanced tumor cell apoptosis	CD90 expression in different concentrations (CD90^high^ ADSCs and CD90^low^ ADSCs) on ADSC-EVs affected the antitumor activity	*In vitro*	[40]
BM-MSC	Suppressed the growth of triple-negative breast cancer	By secreting miR-106a-5p	*In vivo*	[41]
hUC-MSC	Promoted the invasion and migration potential of breast cancer cells	By activating the Akt pathway to promote epithelial-mesenchymal transition	*In vitro*	[36]
hMSC or mMSC	Promoted the progression of breast cancer	By inducing monocytic myeloid-derived suppressor cells to differentiate into highly immunosuppressed M2 polarized macrophages	*In vivo*	[42]

MSC: mesenchymal stem cell; EV: extracellular vesicle; ADSC: adipose-derived MSC; BCC: breast cancer cell; MSC-EV: MSC-derived EV; ADSC-EV: ADSC-derived EV; BM-MSC: bone marrow MSC; hUC-MSC: human umbilical cord MSC; hMSC: human MSC; mMSC: mouse MSC.

**Table 2:** Effects and mechanisms of MSC-EXs on GC.

Source	Effect	Mechanism	Model	Ref
p53^−/−^ mBM-MSC	Promotion of the growth and metastasis of gastric cancer	Delivery of UBR2 to p53^+/+^ mBM-MSC and MFC cells by modulating the Wnt/*β*-catenin pathway	*In vitro*	[48]
BM-MSC	Promotion of the growth of osteosarcoma (MG63) and GC (SGC7901) cells	Activation of the Hedgehog signaling pathway	*In vitro*	[49]

MSC: mesenchymal stem cell; p53^−/−^ mBM-MSC: p53 deficient mouse bone marrow MSC; p53^+/+^ mBM-MSC: p53 wild-type mouse bone marrow MSC; MFC: murine foregastric carcinoma; BM-MSC: bone marrow MSC; GC: gastric cancer; hUC-MSC: human umbilical cord MSC.
